# Supplementary material for: Ferric Carboxymaltose in Patients with Acute Decompensated Heart Failure and Iron Deficiency: A Real-Life Study
Source: J Pers Med. 2023 Aug 12;13(8):1250. doi: 10.3390/jpm13081250 (PMC10455601; doi:10.3390/jpm13081250)
Supplement: Supplementary file 1 [file jpm-13-01250-s001.zip › jpm-2515953-supplementary.pdf]

Supplemental Table S1. Ferric carboxymaltose dosing schedule.

	Body weight <70 Kg			Body weight ≥70 Kg		
**Hb (g/dL)**	< 10	10-14	>14 <15	< 10 g/dL	10-14	>14 <15
**Target dose**	1000 mg	1000 mg	500 mg	1000 mg	1000 mg	500 mg

Hb: Hemoglobin

Supplemental Table S2. Follow-up results according to LVEF

	LVEF≤50% (n=35)	LVEF>50% (n=44)	p-value
Death, n (%)	8 (22.9)	5 (11.4)	0.3085
All cause readmissions, n (%)	24 (68.6)	29 (65.9)	0.9432
Readmissions for heart failure or cardiovascular events, n (%)	16 (45.7)	21 (47.7)	0.7695
LOS (days), median (Q1, Q3)	9 (7, 14)	9 (7, 11)	0.6746

EF: ejection fraction; LOS: length of stay.

Supplemental Table S3. Follow-up results according to FCM supplementation and LVEF

	FCM supplemented	FCM unsupplemented	p-value
LVEF≤**50%**			
Death, n (%)	4 (20.0)	2 (25.0)	1.0000
All cause readmissions, n (%)	12 (60.0)	4 (57.1)	1.0000
Readmissions for heart failure or cardiovascular events, n (%)	8 (40.0)	1 (14.3)	0.3632
LOS (days), median (Q1, Q3)	9 (8, 15)	8 (5, 11)	0.1469
LVEF>**50%**			
Death, n (%)	5 (17.9)	0 (0.0)	1.0000
All cause readmissions, n (%)	20 (66.7)	6 (85.7)	0.6486
Readmissions for heart failure or cardiovascular events, n (%)	16 (53.3)	4 (57.1)	1.0000
LOS (days), median (Q1, Q3)	11 (8, 15)	6 (3, 6)	0.0002

EF: ejection fraction; LOS: length of stay.

Supplemental Table S4. Follow-up results according to LVEF among FCM supplemented patients

	LVEF≤50% (n=22)	LVEF>50% (n=31)	p-value
Death, n (%)	4 (20.0)	5 (17.9)	0.8513
All cause readmissions, n (%)	12 (60.0)	20 (66.7)	0.6304
Readmissions for heart failure or cardiovascular events, n (%)	8 (40.0)	16 (53.3)	0.3552
LOS (days), median (Q1, Q3)	9 (8, 15)	11 (8, 15)	0.9282

EF: ejection fraction; LOS: length of stay.

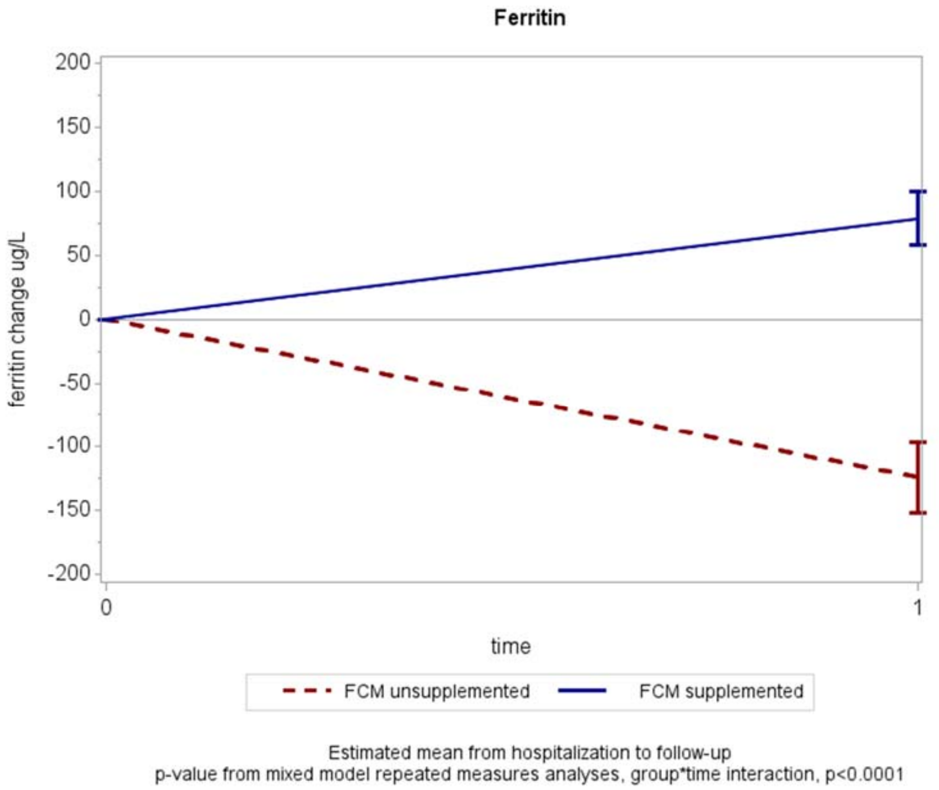

Supplemental Figure S1: Changes from baseline (time 0) to follow-up (time 1) of ferritin levels in FCM supplemented (solid line) and unsupplemented patients (dashed line)

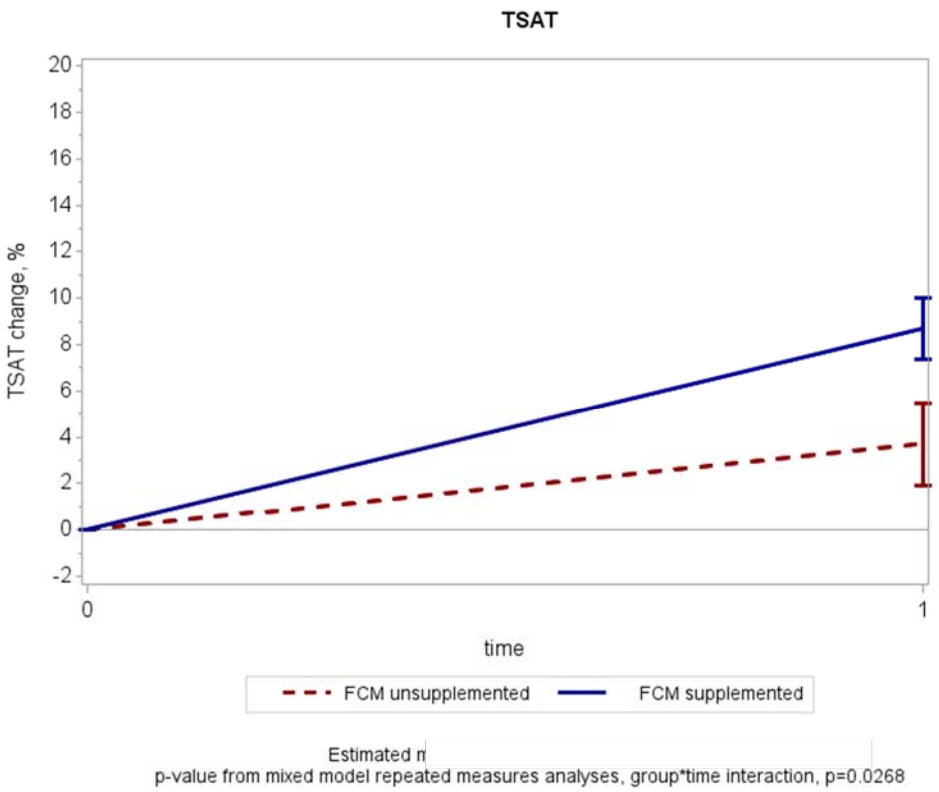

Supplemental Figure S2: Changes from baseline (time 0) to follow-up (time 1) of TSAT levels in FCM supplemented (solid line) and unsupplemented patients (dashed line)
(see attachments)
